# The impact of severe asthma on patients’ autonomy: A qualitative study

**DOI:** 10.1111/hex.12879

**Published:** 2019-03-21

**Authors:** Daniela Eassey, Helen K. Reddel, Kath Ryan, Lorraine Smith

**Affiliations:** ^1^ School of Pharmacy, Faculty of Medicine and Health University of Sydney Sydney NSW Australia; ^2^ Woolcock Institute of Medical Research University of Sydney Sydney NSW Australia; ^3^ School of Pharmacy University of Reading Reading UK

**Keywords:** autonomy, qualitative study, self‐determination theory, severe asthma

## Abstract

**Background:**

People living with severe asthma may have ongoing debilitating symptoms despite high‐dose treatment. Clinical guidelines for severe asthma recommend concepts such as patient centeredness, shared decision making and self‐management, at the heart of which lies autonomy.

**Objective:**

This study aimed to explore the role of autonomy in patients’ narratives about their experiences of living with and managing severe asthma.

**Methods:**

In‐depth semi‐structured interviews were video‐ and/or audio‐recorded and transcribed. Data were categorized using a hybrid approach to analysis incorporating both inductive and deductive methods, informed by the self‐determination construct of autonomy. Analysis and comparison across and within categories were conducted to develop final themes.

**Results:**

Twenty‐nine face‐to‐face interviews, lasting 1.5‐4 hours, were conducted across Australia. Patients’ autonomy was enacted or challenged in a range of situations, such as interacting with health‐care providers, maintaining employment, managing symptoms, and dealing with threats to self‐identity. Two main themes were discerned from the analysis: (a) the desire to live an “unconstrained” life; and (b) preservation of self‐identity.

**Conclusion:**

Our findings suggest that autonomy is broader than conventional medical concepts such as decision making and information seeking. Future research should consider these findings when developing and implementing patient‐driven self‐management interventions for those living with severe asthma.

## 1 INTRODUCTION

Asthma is a chronic condition of the lungs, affecting people of all ages.^1^, ^2^ It can range from mild to severe, based on the level of treatment required to control symptoms and prevent asthma attacks. Severe asthma affects 3%‐10% of the asthma population.^1^ The term severe asthma in clinical guidelines is now formalized as asthma that requires high‐dose treatment for it to remain controlled or that remains uncontrolled despite high‐dose therapy.^3^ Severe asthma accounts for high morbidity and is estimated to contribute half the health‐care cost of all asthma.^4^, ^5^, ^6^, ^7^ A study in the United Kingdom reported that, due to frequent hospitalizations and debilitating symptoms, two in three patients living with severe asthma are unable to hold full‐time employment because of their illness.^8^

To improve outcomes for patients with either mild or severe asthma, guidelines emphasize the importance of patient‐centeredness, shared decision making and self‐management. Central to these concepts is autonomy, a term widely used and valued across the health‐care literature with no gold‐standard definition. Most people prefer autonomy over feeling controlled by someone or something.^9^ However, upon acquiring a chronic medical condition, achieving or maintaining personal autonomy might be challenged through the long‐term burden of symptoms, treatment and their psycho‐social impacts.

Whilst most severe asthma research has been devoted to understanding pathophysiology and biomarkers, and testing novel treatments, some studies have been conducted to promote patient autonomy in asthma, and these have reflected an emphasis on increasing autonomy through education and action plans.^10^, ^11^ In the clinical context, autonomy has been defined as the level of involvement patients desire to gain information and make decisions about their asthma.^10^ Theoretically, however, autonomy is broader than information seeking and decision making; research in other fields has been conducted to explore these broader aspects.

According to self‐determination theory (SDT),^12^ autonomy is a concept central to satisfying basic psychological needs for well‐being,^13^ and is viewed as an intrinsic source of motivation to master one's own destiny, to have control over one's life and behaviour, and to feel self‐directed. Importantly, autonomy aligns with one's values and beliefs, and sense of self or identity.^14^, ^15^ Over the past 20 years, autonomy has been explored through studies such as workplace motivation,^16^, ^17^, ^18^ health care,^19^, ^20^ physical activity,^21^ education^22^ and leadership.^23^ Whilst such work has examined and tested the efficacy of clinical interventions for diverse issues related to behavioural change, motivation and supporting autonomy, SDT has not been utilized to analyse people's personal experiences of living with a chronic condition and the role that autonomy plays in their day‐to‐day lives. In the context of a long‐term and debilitating condition such as severe asthma, even daily activities might present a threat to feelings of personal control, choice and decision making. A recent review identified few studies investigating patients’ perceptions of living with severe asthma^24^; these studies focused on the clinical rather than personal aspects of the lived experience of severe asthma. The review highlighted that severe asthma can be disempowering and poses threats to identity and life roles.^24^

The increasing importance and popularity of supporting autonomy in health care,^25^, ^26^ combined with the paucity of exploratory studies of experiences of living with severe asthma, highlight the need for research in this area. Thus, the overall aim of this study was to explore the role of autonomy in patients’ narratives about their experiences of living with and managing severe asthma. As autonomy can be expressed in many forms, we looked for when instances of volition, exercising autonomy and resisting external sources of control (or not) occurred.

## 2 METHODS

### 2.1 Study design

A qualitative interview approach was chosen for this study because it can provide deep insights and offer rich sources of information.^27^ The research protocol was approved by the University of Sydney Human Ethics Committee (HREC 2015/934).

### 2.2 Participants

Potential participants were invited to take part in the study through their general practitioners or respiratory physicians. The aim was to include participants from different ethnicities, geographical locations and sociodemographic backgrounds. They had to be ≥18 years and diagnosed with severe asthma by a specialist respiratory physician. A trained qualitative researcher (DE) conducted the interviews.

### 2.3 Data collection

The researcher conducted the interviews in the respondents’ homes, or elsewhere if preferred. Interviews were collected between October 2016 and May 2018. Interviews were conducted face to face, to explore in depth the individual's experiences and perspectives of living with severe asthma. Participants were asked to tell their own story from the point when they first noticed they had breathing problems. They were encouraged to talk about their experiences of living with this condition, with as little interruption as possible from the interviewer. Interview topics were initially defined from a systematic review of the literature 24 but later modified as new themes emerged. Follow‐up open‐ended questions and prompts were used to further explore participants’ experiences. An overview of the questions asked during the interview is included in Table 1. The interviews were video and/or audio recorded and then transcribed verbatim. To protect the anonymity of the participants, pseudonyms have been used in place of their real names.

**Table 1:** Semi‐structured interview guide‐ sample questions

1. Can you take me back to your first memory of having breathing problems?
2. Do you remember the early days of living with severe asthma (SA)? Tell me about that.
3. What was your reaction to getting a diagnosis of SA? How did you feel about receiving this?
4. What have your experiences been of telling family/friends/colleagues that you have SA?
5. To what extent has living with SA impacted on your life? Tell me about a couple of instances of how this impact has affected your life.
6. Where or from whom do you find you get the most support?
7. What does “support” mean to you?
8. What has your experience been of the medications you have had to take for SA?
9. Tell me what happens when you go to your Dr?
10. How is it decided on what medication/treatment you get?
11. How has the distance geographically been a problem in terms of accessing services?
12. What does “severe asthma” mean to you?
13. How have things changed over the years for you in terms of living with your SA?
14. What are your goals? (life goals? asthma goals?)
15. What do you think your Drs’ goals are?
16. What are your biggest concerns?
17. To what extent do you feel your condition inhibits you from doing the things you want to do?
18. To what extent do you feel you have control over your condition?
19. What does “asthma control” mean to you?

### 2.4 Data analysis

Data analysis was conducted using a hybrid method of thematic analysis, incorporating both data‐driven inductive and SDT‐informed deductive approaches.^14^, ^15^, ^28^ The SDT construct of autonomy informed the development of a template of codes as a means of organizing text for subsequent interpretation.^28^ The transcripts were coded using NVivo software and were read by three members of the research team (DE, LS and KR). During later stages of data collection, the constant comparison method of analysis was applied to continuously compare the views and experiences of participants, and subsequent revision of the coding template.^29^ Themes were then developed by DE and LS. This required stepping into more complex territory whereby themes were discerned from the conceptual codes as well as from relationships between the codes.^30^ The interpretive analysis was then reviewed by researchers with experience in qualitative research (LS, KR and HR) and in the clinical aspects of severe asthma (HR).

## 3 RESULTS

**Figure 1:**
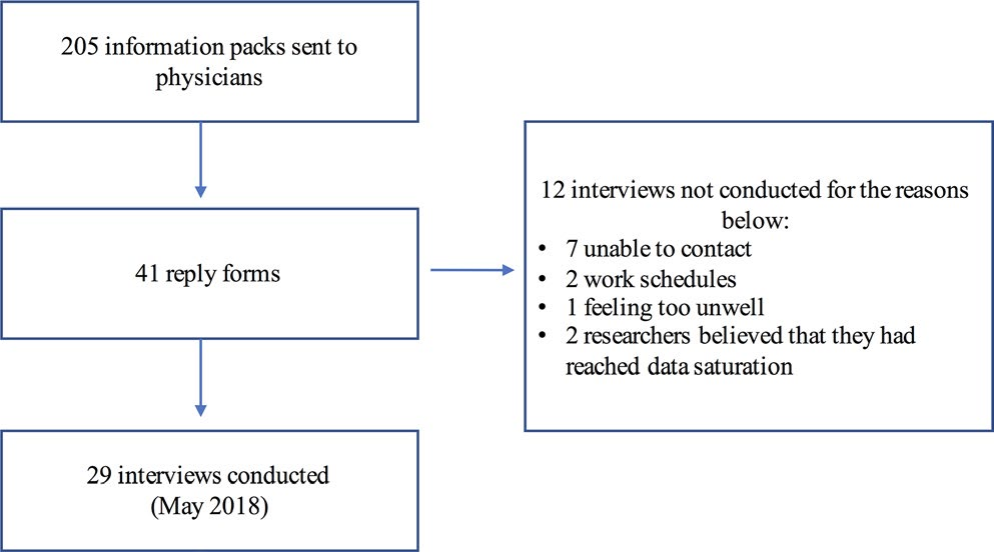
Flow diagram for participants included in study

Overall, 205 participant information packs were sent to respiratory physicians and general practitioners to distribute to their eligible patients: 41 reply forms were sent back to the researcher (DE). Twenty‐nine interviews were conducted as of May 2018 (Figure 1). Most of the participants agreed to being video‐recorded. The interviews were 81‐232 (mean 155) minutes in length. Table 2 shows basic demographic characteristics. All participants spoke English fluently. Eighteen reported having at least a high school education.

**Table 2:** Characteristics of the participants (n = 29)

	N
Age range in years (mean)	19‐74 (52)
Gender	
Female	15
Male	14
Time since diagnosis of severe asthma	
≤10 y	12
>10 y	17
Cultural identity	
Anglo‐Saxon (including Italian, German, Irish or British)	27
Asian	1
Persian	1
Geographical location	
Metropolitan	18
Regional	7
Rural	4
Employment status	
Employed	10
Retired	11
Unemployed	8

Our findings show that autonomy was enacted or challenged in a range of situations, such as interactions with health‐care professionals (HCPs), managing symptoms and medications, employment and challenges to identity. Above all, individuals endeavoured to obtain control over their condition and valued their autonomy: *I wasn't going to let it beat me. I wasn't going to give in and there were times when I could've… (Richard, 54‐year‐old man)*.

**Figure 2:**
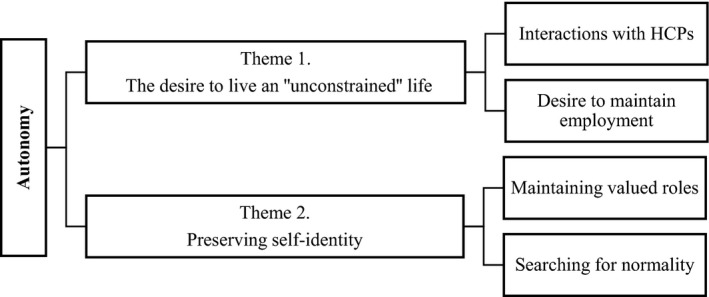
Conceptual diagram of the overarching theme and subthemes disseminated in this study

Our analysis revealed two themes illustrating the ways in which autonomy was enacted, or challenged, in people's experiences of living with severe asthma. These were as follows: (a) *the desire to live an “unconstrained” life*; *and (b) preserving self‐identity *(Figure 2).

### 3.1 Living an “unconstrained” life

Participants described at length how the debilitating effects of their condition, and how expectations of self and others, disrupted their everyday lives and challenged their autonomy. A strong desire to live an *“unconstrained” *life emerged, reflected in two key sources of autonomy: first, through their interactions with HCPs; and second, through their desire to maintain employment.

#### 3.1.1 Health‐care interactions

Making decisions and choosing courses of action regarding their health were most evident amongst participants’ experiences with health‐care interactions and managing medications. Overall, exercising autonomy was described as being empowering and reassuring, although sometimes autonomy was exercised even when the outcome was unwanted. At times, participants took steps to actively oppose perceived controlling efforts by others, such as HCPs, even if this might not have been in the best interests of their health.

Individuals living with severe asthma valued having a sense of choice over being told what to do and feeling heard by their HCPs. Some described that they needed to feel like they were given a choice and that their values and beliefs were taken into account, before personally endorsing any health‐related behaviours. They also highlighted the importance of self‐initiating and regulating these behaviours. Jane reported: *Never give up…If you think there's something wrong, that someone's not saying, speak up. You've seen something [health‐related issue that her healthcare providers didn't pick up] obviously but they didn't take the time to see … speak up. (Jane, 51‐year‐old woman).*

Autonomy was exercised, and challenged, through a range of health‐care interactions. Participants felt empowered when their HCPs asked what was important to them and gave them opportunities to make choices over health‐related decisions. As Sarah stated, being able to make choices helped to thwart the helplessness of hospitalizations:

…the same doctor asked me the same questions on three of my admissions, ‘what do you need?’ And it was like, I need this, this and this… It makes me powerful. I think when you go to hospital you basically give away all your power a lot of the time. Not because you want to but because you’re too sick to bother. So, when you get given it back it’s like you’ve got your power back now, … you’re in charge. (Sarah, 59‐year‐old woman)

Having input into treatment options and knowing about medications and their side‐effects were empowering. To be reliant on health professionals and uninformed was viewed as disempowering:

…one’s empowering and the other one’s totally disempowering. It sort of means, I’m relying on the fact that you [doctor] know what you’re doing and if you don’t, then I don’t have a say anyway and sometimes I feel like, well, I think we need to discuss what the side effects are and why I’m taking it. (Sarah, 59‐year‐old woman)

Being unable to breathe was described as fear‐provoking, challenging their sense of personal control: “*…it's more fear than anything else. Like oh this is bad… yeah that's not a good feeling at all. Especially when you know that like the medication I've taken and that wasn't working.” (Ben, 60‐year‐old man)*. At times, the memory of having an asthma attack was a strong motivator to engage in health‐related behaviours and exert personal control. Health‐related behaviours, such as adhering to treatment, increasing physical activity and avoiding triggers, were valued as a way of maintaining a sense of control over severe asthma. For example, Chris reported that he would keep a six‐month supply of his medication as this helped him feel like he was in control of his body; he saw not having plenty of medications as a potential threat to his sense of personal control:

I’m out of control, I start to worry. But if you’ve got everything there you need to control it, you don’t worry…nothing worse than not being in control of your body, not being able to breathe… (Chris, 66‐year‐old man)

Access and adherence to prescribed medications were perceived as enablers to gaining a sense of control over one's life. For example, Jane had a system which provided reassurance that she could carry out her daily activities:

I call it lock and load [a term originally used in relation to firearms, later popularised by a John Wayne movie set in World War II] and I normally have one puffer in here [in one pocket] and one puffer in there [in the other pocket]. (Jane, 51‐year‐old woman)

Exercising autonomy, however, in some cases could also symbolize the ultimate loss of personal control and choice. Phil reported that it took him time to come to terms with the diagnosis. He did not understand the severity of his condition, and despite his HCP's advice to not push himself physically on the farm as this may trigger an asthma attack, he exercised autonomy, and as a result of this, he was hospitalized:

Just couldn't‐couldn't believe it [being diagnosed with severe asthma], I was just super human. I just didn't think, that could ever happen to me…We have a fair few grapes, they're virtually picked at night‐time…[and] it started to get really, really bad. So, I… barely got home and went into the hospital, and, they put me in intensive care. (Phil, 74‐year‐old, man)

Whilst avoiding triggers and adhering to medications helped to provide a sense of control over severe asthma for some participants, for others different aspects of their lives were more highly valued. Asserting autonomy to preserve these, despite the potential health problems that might arise as a result, was evident. For example, Grace reported that her specialist told her not to keep her pet budgerigar (native Australian bird). However, for her this was not an option, as the budgie was part of her family:

I was told that I was to not have budgies because they carry bird fancier’s disease. Yeah but his cage gets kept clean and he runs the house. So, what my specialist doesn’t know won’t hurt me. (Grace, 41‐year‐old woman)

Rich narratives on the importance of feeling understood, heard and valued by HCPs mainly related to events when participants felt that their views were not considered. In these instances, autonomy was expressed through active “pushing back” behaviours as a way of re‐asserting control. Some reflected on a time when their views conflicted with their physicians’ view of what treatment was best for them, and as a consequence took action to assert their autonomy.

My first specialist said ‘oh your condition is bad’, and his reaction was to stick me on cortisone medication straight away and…I went back to my GP and said look I wasn’t really comfortable with him and I asked to be referred to another one. (Dylan, 65‐year‐old man)

The need to feel recognized by one's doctor as an expert on living with severe asthma was also valued, as was having an equal partnership. Mel expressed that her doctor would become “*aggro*” every time she was hospitalized due to her asthma, and he suggested that she should go into a nursing home. She felt that her views were not considered, and one way of exercising her autonomy was to “train” her doctor:

I’m training him [general practitioner]. Um, certainly not an equal partnership no but…‐‐ I‐I tell people I'm trying to train him and to be quite honest I think I am. I think there is a lot of things he doesn’t understand…they don't like anyone that is an expert over them…we are the ones that live with it. (Mel, 66‐year‐old woman)

#### 3.1.2 Employment

Maintaining employment whilst living with severe asthma was a highly valued aspect of life, but the debilitating effects of the condition were a constant threat to people's capacity to work. Many examples emerged of participants pushing their physical limits, improving fitness, planning ahead, and resisting perceived controlling efforts by others. These autonomous actions helped to preserve a sense of productivity and contribution to society and maintained social ties. Sometimes severe asthma “won,” and employment was no longer an option. Dylan reported that there were days he should have stayed home due to his condition, but he decided to push through and go to work*:*

I never took as much sick leave as I should have unfortunately. I left the bank with over a year’s worth of sick leave accumulated. Being manager of the department, I felt I had to be there as often as possible and I know I should not have been there certain days. (Dylan, 65‐year‐old man)

Other participants viewed going to work as not allowing severe asthma to control their lives. Joseph reported that even when his severe asthma was flaring up, this did not stop him from going to work:

I am a very hardworking person. Normally even when I am sick, next morning I’ll try to do my job, because I’m a very responsible person…. I feel if I stay at home and do nothing, I feel worse, even my body becomes worse. (Joseph, 70‐year‐old man)

Threats to individuals’ autonomy were also evident and emerged as not being able to have a choice and feeling like the condition had intruded in their life. For example, Jane reported that work (physiotherapist) was a big part of her life to the point that despite regular hospitalizations she had her uniform in the car and would still go to work. It was *getting incredibly incredibly difficult… [and I had] to reduce the types of patients [I] was seeing*, she had to turn down patients who came in sick, so work was *much more of a risk*. Eventually, she had to stop working and *life became very reduced*.

Despite HCPs’ recommendations to cease work in a participant's preferred occupations, participants resisted these external efforts to control. Hugh (chef) reported that his HCP told him to give up work, but he felt that this advice *was kind of belittling…it was kind of insulting when he said, you know, you can't do this, you need to be more realistic *(*Hugh, 19‐year‐old man)*. Despite acknowledging that he lives with an unpredictable condition and recognizing his specialist's recommendations, he was not prepared to give up his profession and responded with: *I'll take my chance*. Acting autonomously, even when that involved resisting controlling efforts of expert others, and the possibility of poor health, enabled this young person to have a sense of purpose.

### 3.2 Preserving self‐identity

Autonomy was enacted and challenged at times to preserve “self‐identity.” For some, having their identity embedded within the illness provided periods of psychological vulnerability and loss of personal control. This was often due to severe asthma challenging the unity between the body and self. At times, participants described that when they felt their identity confronted by the illness they would “push back” to limit the effects of the illness on self. Self‐identity was represented by being able to maintain valued roles, preserve their sense of “normality,” or for some, search for a “new normality.”.

#### 3.2.1 Maintaining valued roles

Notions of self‐identity, maintaining valued roles and autonomy were inextricably linked. The debilitating effects of severe asthma were a constant threat to people's sense of identity, yet there was a strong desire to maintain valued roles. Many examples emerged of individuals not surrendering their self to the illness and, instead, finding ways to preserve their roles. For example, Donna identified herself as a mother of two daughters, however, being in and out hospital due to severe asthma challenged her identity as she felt she could not always fulfil the role of being a mother.

You’ve got kids that want to play with you, and you can’t. And the worst thing has been the amount of times I have been in hospital when it’s my kids’ birthdays or I’ve just been too sick… we try and make up for it, you know, I buy way too much stuff to try and make up for it…our big purchase was we bought them a trampoline, and that cost a fortune. Thank you credit card. (Donna, 39‐year‐old‐woman)

Another participant, Phil, a third‐generation farmer who owned eight farms, valued the ability to preserve a multigenerational family legacy whilst living with severe asthma. He compared his HCPs as experts in their field to him being the expert in farming *…they're the professionals, they're the equivalent of Phil of farming, I know anything in farming…. *He spoke at length about how he would always preserve his identity of being a farmer and would not let his condition get in the way: *I started when I was 15, so. Uh, just farmed all me life… I'm doing what I love to do, and, uh, the alternative to that, take it away from me, and I definitely will die. So, there's no way I'm going to give up what I'm doing. (Phil, 74‐year‐old, man)*.

#### 3.2.2 Searching for normality

Receiving the diagnosis of severe asthma was overwhelming and challenged participants’ autonomy and sense of identity. Participants recounted working towards preserving parts of their life that had preceded the diagnosis of severe asthma, including engaging in activities and socializing. Exercising autonomy was tied up with acquiring a sense of volition and preserving a sense of “normality.” Jane described that, prior to being diagnosed with severe asthma, she defined having a “normal” life as being an active person, enjoying the outdoors and being independent: *I was living like normal…. I used to dance. I used to pick fruit. I used to mow lawns. I used to be able to take care of myself. *Since being diagnosed with severe asthma, she described struggling to define her identity. However, one way of exercising her autonomy was by being *realistic* and, rather than surrendering to the illness, preserving normality by doing things at a slower pace:

I’ll be realistic …being able to travel, to be able to go fishing, being able to cook around the campfire, being able to look after myself, still being able to help people that’s been a big thing for me and I've lost that…I've got to do something whether it be you know I might try to cook dinner and fail at it but if I don’t try, what am I going to do? Sit here and mourn away. (Jane, 51‐year‐old woman)

Autonomy was also enacted when trying to search for a new normal. Resetting normality required negotiation and reconceptualization of a normal life by incorporating the illness. For example, Richard stated that because he had severe asthma, his father, a farmer, had to sell the farm: *It would much have affected our whole family. I should have been a fourth‐generation farmer. So, it changed our whole family.* This challenged his identity, and at times, he compared what he perceived as a “normal life” to the way he now lived. The shift in his identity and the struggle for self‐preservation involved an ongoing process of negotiation to find a new “*normal*.”.

A normal life would be being…able to work in a normal job for the rest of your life, being able to afford to pay your mortgage, being able to afford to do all those things that money comes with. I’ve still got as normal a life as I can. I've got friends, I've travelled, I've got my home, which I'm still paying… I had to beat it to still be here. I've still got it [severe asthma], but it's like we've reached an agreement. (Richard, 54‐year‐old, man)

Efforts to preserve “*normality”* had an impact on emotional, physical and mental health. Individuals expressed that, despite their illness, they had been able to maintain autonomy by doing things such as home chores or gardening at a slower pace, but at a physical and emotional cost. Archie still attempted to accomplish physically demanding tasks to maintain autonomy. This resulted in him feeling both physically weak and emotionally drained:

I just want to be left alone. I just want to sit in the corner of the couch and, and I’ll sit there for a week…I helped the guy next door…he didn’t know how to build a little retaining wall…I was just sopping sweat and he goes, ‘what’s wrong with you?” And like I was just dripping wet and couldn’t breathe and just using the, the puffers. And it wasn’t a, a big job to do. But… so you’re just embarrassed… (Archie, 58‐year‐old, man)

## 4 DISCUSSION

The purpose of this study was to examine in depth the role of autonomy amongst those living with severe asthma. To the author's knowledge, this study has provided the first insights into the role of autonomy in people's experiences of living with this condition. We argue that autonomy was challenged and enacted in efforts to: (a) *live an “unconstrained” life:* this was influenced by participants’ interactions with HCPs and desire to maintain employment; and (b) *preserve self‐identity: *participants sought to maintain their valued role and strived for “normality.”

In the words of Dworkin,^31^ autonomy means endorsing one's actions at the highest level of reflection; this is one of the main defining aspects of autonomy in SDT. It is crucial to autonomy that individuals feel that they can raise questions and identify or reject reasons for which they will act upon a decision, with no external influences such as manipulation or pressure from anyone else.^31^ Our findings highlight the challenges people living with severe asthma face to preserve their autonomy to live an *“unconstrained” *life. One such challenge was how individuals described the importance of having a sense of choice over being told what to do, and feeling heard by their HCPs. Previous research has also uncovered various threats to autonomy such as HCPs’ often paternalistic approach to treatment, both in severe asthma^24^ and in chronic illness in general.^32^ Similarly, patients with other chronic conditions also appreciate when HCPs support their personal autonomy.^33^ Naik et al^34^ expanded the concept of autonomy and recommended strategies to support patients who struggle with “executive autonomy”—the capacity to carry out treatment plans. Self‐determination theory research has highlighted the importance of supporting patient autonomy in health‐related decisions.^9^, ^12^, ^13^, ^19^ According to Deci and Ryan et al,^13^ this improves motivation for making health‐related behaviour changes. Health professional support for autonomy by empowering patients with severe asthma, hearing their concerns and giving them choice helps thwart feelings of powerlessness and helplessness.

Current guidelines suggest broadening the traditional biomedical approach of health care by encompassing patients’ needs and values.^35^ Our study has highlighted that the use of narratives can help to further understand the impact of severe asthma on patients' experiences of personal control, choice and decision making. Previous studies examining health‐care workers’ views on practising holistically reported barriers to involving patients, including time constraints.^36^, ^37^, ^38^ However, according to Kalitzkus et al,^39^ allowing a narrative flow in the consultation enables self‐reflection on daily practice and improves patient‐physician relationships without necessarily requiring a lot of time.^39^

Whilst adhering to medications and feeling supported by HCPs provided a sense of control over severe asthma for some participants, other valued aspects of their lives could take precedence. Our findings highlight that maintaining employment whilst living with severe asthma was a highly valued aspect of life. Research has shown that people living with chronic illnesses continually negotiate autonomy in their daily activities and in their relationships.^32^, ^40^ Our study participants would push their physical limits, plan ahead and resist any perceived controlling efforts by others in order to live a life where they could either maintain employment or preserve their sense of productivity. A UK study reported that due to the unpredictable nature of severe asthma, two‐thirds of patients were unable to hold full‐time employment.^8^ However, our findings go beyond those of previous studies that describe the impact of severe asthma on an individual's life^8^, ^24^, ^41^ and demonstrate how autonomy was enacted by people with severe asthma to preserve a sense of productivity and contribution to society.

This study has important clinical implications for HCPs in identifying and understanding the day‐to‐day psychological and physical challenges patients face to support self‐management and maximize quality of life. The constant challenge of having difficulty breathing despite adhering to treatment triggered strong emotions such as fear and panic. It became clear that participants were constantly battling the tension between both exerting and losing personal control. On the one hand, the memory or anxiety associated with having an asthma attack was a motivator to exert control and engage in specific health‐related behaviours to control symptoms. On the other hand, the experience of severe breathlessness represented a loss of personal control, resulting in fear‐provoking emotions. Studies have shown that anxiety and depression are common amongst people living with severe asthma.^42^, ^43^, ^44^ A study by McDonald et al^45^, ^46^ reported that anxiety was 1.4 times more common, and depression 3.3 times more common, in people with severe compared with non‐severe asthma.

Our findings highlight that, despite challenges to autonomy, patients had a strong desire to preserve their identity. This resonates with the work by Corbin and Strauss,^47^ Bury^48^ and Charmaz^49^ on biographical disruption, where they describe that living with a chronic illness assaults the body and disrupts a sense of wholeness of self. Our study highlights that for some, in the context of biographical disruption, the onset of severe asthma brought about a “major kind of disruptive experience.”^48^ For others, however, it triggered autonomous actions to preserve or reconstruct their identity as a means of reorientating their lives. Furthermore, enacting autonomy by incorporating severe asthma fully into their identity was a way to maintain or reset normality. Moreover, looking at the narratives of people's experiences of living with severe asthma through the lens of autonomy has the potential to further our understanding of the complex ties between the experiences that embody the shifts in identity and the psychological efforts required to unite the body and self.

Preserving the “self” in severe asthma becomes harder when there is a constant struggle to fight the debilitating physical effects of the illness and side‐effects of its treatment, and the identifications that come with the diagnosis. When trying to maintain and preserve “self” in the face of illness, individuals acknowledged the emotional cost associated with their efforts in preserving “normality.” Previous quantitative and qualitative researches on patients’ experiences with severe asthma note that its debilitating and unpredictable symptoms, the side‐effects of treatment and the burden on their quality of life can ultimately have emotional consequences,^41^, ^50^, ^51^ but there is a lack of in‐depth psychological research furthering our understanding of *how* people living with severe asthma perceive their condition and *how *they behave when challenged in a range of situations. Our findings attempt to broaden and challenge the biomedical model, and highlight that asserting autonomy to preserve their sense of “normality” and sense of self might also lead to unwanted outcomes. This suggests that exploring the individual's perspective could be used to further understand the psychological impact of living with severe asthma.

## 5 STRENGTHS AND LIMITATIONS

Strengths of the study include its rigorous qualitative methodology and the use of a theoretical frame of reference. The interviews were conducted face to face to build a relationship, and to facilitate openness and in‐depth discussions. Using a narrative illness approach, participants controlled the structure, length and content of the interviews. Their stories showed how people living with severe asthma accounted for their individual experiences of illness and health care. This revealed a range of significant psychological, emotional and physical elements of living with a debilitating condition. There are several limitations to acknowledge. First, our participants did not reflect the ethnic diversity in the Australian population; however, this might reflect access to specialist care, as 85% of patients registered in the Australian Severe Asthma Web‐based Database were Caucasian.^52^ In addition, qualitative researchers do not aim for generalizability but for transferability,^53^ that is parallel to external validity or the extrapolation of findings to similar situations. Second, participants were invited to take part in the study through their general practitioners or respiratory physicians. This could have influenced patient selection; however, our study criteria included the current international guidelines definition of severe asthma,^3^ which requires investigation by a clinician to exclude common modifiable causes of uncontrolled asthma. To counter possible influence, prior to the interview, participants were advised that what was said during the interview would not be shared and would have no effect on their relationship with their health‐care providers.

## 6 CONCLUSION

This study has advanced our knowledge in understanding the complex dynamics of patient autonomy in contemporary medical practice, using the narratives of people living with severe asthma. Using the SDT as a framework allowed us to question assumptions made within the biomedical model about people's experiences of living with severe asthma and their associated behaviours. It has improved our understanding by identifying alternative ways of supporting patient's self‐management. Given that the majority of research aimed at developing policy and practice for severe asthma describes the need to promote patient autonomy, our findings suggest that autonomy is broader than the conventional concepts in asthma literature of decision making, information seeking, symptom management and adherence to treatment. Participants demonstrated that autonomy was enacted to maintain their values and beliefs, as well as during the process of preserving their self‐identity. The findings of this study should be considered when developing and implementing patient‐driven self‐management interventions for those living with severe asthma.

## AUTHORS’ CONTRIBUTION

All authors contributed to the conception and design of the study. DE was involved in acquisition of data. All authors performed analysis and interpretation of data. DE drafted the article. All authors were involved in revision and final approval of the article.

## ETHICAL STATEMENT

The research protocol was approved by the University of Sydney Human Ethics Committee (HREC 2015/934).
